# Human bone marrow mesenchymal stem cells-derived exosomes attenuated prostate cancer progression via the miR-99b-5p/IGF1R axis

**DOI:** 10.1080/21655979.2021.2009416

**Published:** 2022-01-14

**Authors:** Shichun Jiang, Haiyu Chen, Kai He, Juan Wang

**Affiliations:** a Department of Urology, Mianyang Central Hospital, Mianyang City, Sichuan Province, PR. China; b Department of Surgery, Haikou Hospital of Traditional Chinese Medicine, Haikou City, Hainan Province, PR. China

**Keywords:** Prostate cancer, HBMSCS, exosome, miR-99b-5p, IGF1R

## Abstract

MicroRNA-99b-5p (miR-99b-5p) has been shown to be enriched in serum exosomes of prostate cancer (PCa) patients treated with radiotherapy, while its function in PCa progression remains unclear. The expression levels of miR-99b-5p in PCa tissues, cancer cell lines and human bone marrow mesenchymal stem cells (HBMSCs), as well as HBMSCs-derived exosomes were assessed by quantitative real-time PCR (qRT-PCR). MiR-99b-5p mimics or inhibitor was transfected into HBMSCs, and HBMSCs-derived exosomes with abnormal expression of miR-99b-5p were used to stimulate PCa cell-line LNCaP cells. Cell proliferative rate was evaluated using Cell Counting Kit-8 (CCK-8) and 5‐ethynyl‐2′‐deoxyuridine (EdU) staining assays. Cell migration and invasion were analyzed by Transwell assay. The epithelial-mesenchymal transition (EMT) was evaluated by detecting EMT-related markers using Western blot analysis. The animal model was constructed to confirm the function of miR-99b-5p *in vivo*. The expression levels of MiR-99b-5p were decreased in PCa tissues and cell lines, while elevated in HBMSCs-derived exosomes. HBMSCs-derived exosomes significantly inhibited cell malignant phenotypes of PCa cells, and miR-99b-5p mimics transfected HBMSCs further enhanced the inhibitory effects of HBMSCs on PCa progression. In addition, miR-99b-5p inhibitor transfected HBMSCs-derived exosomes promoted the progression of PCa *in vitro*. Insulin-like growth factor 1 receptor (IGF1R) was identified as a downstream target of miR-99b-5p. Moreover, miR-99b-5p mimics transfected HBMSCs obviously inhibited tumor progression by downregulating IGF1R in animal model *in vivo*. Our results demonstrated that HBMSCs could attenuate PCa progression, and exosomal miR-99b-5p and IGF1R participated in the regulatory process, contributing to our understanding of the pathogenic mechanism of PCa.

## Introduction

Prostate cancer (PCa) is becoming a common and serious malignancy in men worldwide [1]. Even though the overall survival of localized PCa patients is high, metastatic PCa is still largely incurable even after receiving intensive multimodal therapy [2]. Exosomes, secreted from various cell types including reticulocytes and stem cells, are membrane-bound vesicles between 30 and 120 nm [3]. Recently, mesenchymal stem cells (MSCs)-derived exosomes are reported to have the function of tissue repair and the potential for cancer treatment [4]. Exosomal microRNAs (miRNAs) are a type of small molecules with approximately 22 nucleotides in length and exert important regulatory functions in human diseases [5]. Therefore, a better understating of exosomal miRNAs that mediated cancer progression is necessary, which may provide the potential therapeutic insights into cancer treatment including PCa.

MiRNAs lack protein encoding capacity [6], but have been demonstrated to regulate gene expression via binding to the 3ʹ-untranslated region (3ʹ-UTR) of their target genes followed by the inhibition on translation [7]. Exosomal miRNAs have attracted more attentions due to their endogenous characteristics and drug delivery capacity [8]. MiR-99b-5p was shown to participate in cancer progression. It suppresses the progression of cervical cancer by activating the PI3K/AKT/mTOR signaling pathway [9]. MiR-99b-5p was also identified as a diagnostic biomarker in colorectal cancer with abnormal expression [10]. Recently, Malla *et al*. found that miR-99b-5p was notably enriched in serum exosomes of PCa patients after receiving radiotherapy [11], indicating that upregulation of miR-99b-5p might contribute to the treatment of PCa. However, the underlying molecular mechanisms remain unclear.

Increasing evidence has demonstrated that miRNAs exert their essential regulatory functions through targeting downstream genes [12]. One previous study reported that insulin-like growth factor 1 receptor (IGF1R) was a downstream target of miR-99b-5p, and mediated the effect of miR-99b-5p on cancer progression [13]. Recently, IGF1R was also reported to be obviously upregulated in PCa tissues and involved in the development of PCa [14].

In the present study, we hypothesized that hBMSCs-derived exosomal miR-99b-5p might play potential roles in the development of PCa, and aimed to investigate the regulatory axis of exosomal miR-99b-5p and IGF1R in PCa. Our findings revealed that HBMSCs-derived exosomal miR-99b-5p could attenuate the progression of PCa both *in vitro* and *in vivo* by regulating IGF1R, suggesting that hBMSCs-derived exosomes might be a novel therapeutic strategy for PCa.

## Materials and methods

### Tissue samples

A total of 60 tissue samples including 30 tissue samples from prostate cancer (PCa) patients and 30 tissue samples of controls from benign prostatic hyperplasia (BPH) patients were collected at Haikou Hospital of Traditional Chinese Medicine between February 2016 and December 2020. All participants signed the written informed consent. The samples were stored at −80°C before use.

### Cell culture, treatment and transfection

The PCa cell lines (LNCaP, DU145 and PC‐3) and the human normal prostate epithelial cells WPMY‐1 were purchased from American Type Culture Collection (ATCC, Manassas, VA, USA). All cell lines were cultured within RPMI-1640 medium. Cell culture was maintained in an incubator at 37°C with 5% CO_2_. To evaluate the impact of HBMSCs on PCa cells, 20 ug HBMSCs-derived exosomes were treated with LNCaP cells for different time periods, with PBS was used as the control group. To manipulate the expression level of miR-99b-5p, miR-99b-5p mimics, inhibitor and corresponding negative controls (miR-NC and inhibitor NC) were synthesized by GenePharma Co., Ltd. (Shanghai, China) and transfected into PCa cells or HBMSCs using Lipofectamine 2000 (Invitrogen). The sequences were as follows: miR-99b-5p mimic: 5′CACCCGUAGAACCGACCUUGCG-3′ and miR-NC: 5′- UUCUCCGAACGUGUCACGUTT-3′.

### The isolation of HBMSCs, exosomes, and the identification of HBMSCs-derived exosomes

HBMSCs were isolated from young or middle-aged patients with limb fracture and hip replacement (with exclusion of patients with hematological diseases) at the Department of Surgery, Haikou Hospital of Traditional Chinese Medicine as previously described [15]. The morphological characteristics of HBMSCs were observed under an inverted microscope. HBMSCs-derived exosomes were isolated from miR-99b-5p mimics/inhibitor transfected or un-transfected HBMSCs using the ExO-Quick kit (SBI Biosciences, Palo Alto, CA, United States) as previously described [16]. The isolated exosomes were observed under a JEM-1230 electron microscope. Meanwhile, the expression of exosomes surface-related markers CD63, Hsp70, Tsg101 and negative markers GM130 were detected by Western blot analysis.

### Luciferase reporter assay

The potential targets of miR-99b-5p were predicted using the prediction website microRNA.org, and the putative binding sites were predicted by Targetscan [17]. The sequence of 3ʹ-UTR of IGF1R containing the wild type (WT) or mutant (MUT) miR-99b-5p binding site were synthesized by GenePharma Co., Ltd. and cloned into luciferase reporter vector pMIR (Promega). Then the luciferase vectors were co-transfected with miR-99b-5p mimics or miR-NC into LNCaP cells using Lipofectamine 2000. After transfection for 48 h, the luciferase activity was detected.

### Quantitative real-time PCR (qRT-PCR)

Total RNAs of tissue samples and cells were isolated using TRIzol reagent. After reverse transcription, qRT-PCR reactions were conducted using SYBR Green PCR Master Mix (Roche) on an Applied Biosystems 7900 Real-Time PCR System. The relative expression levels were calculated using the 2^−ΔΔCt^ method [18], with glyceraldehyde-3-phosphate dehydrogenase (GAPDH)and U6 small nuclear RNA (snRNA) as the internal references. The primer sequences were as follows: GAPDH, forward: 5′-GGAGCGAGATCCCTCCAAAAT-3′ and reverse: 5′-GGCTGTTGTCATACTTCTCATGG-3′; IGF1R, forward: 5′-TTTCCCACAGCAGTCCACCTC-3′ and reverse: 5′-AGCATCCTAGCCTTCTCACCC-3′; U6, forward: 5ʹ-GCTTCGGCAGCACATATACTAAAAT-3ʹ and reverse: 5ʹ-CGCTTCACGAATTTGCGTGTCAT-3ʹ; miR-99b-5p, forward: 5′-CACCCGTAGAACCGACCTT-3′ and reverse: 5′-TGCACTGGATACGACCGCAAGG-3′.

### Western blot analysis

Western blot analysis was performed as previously described [19]. Total proteins were extracted using RIPA lysis buffer. Protein samples were separated by 12% SDS-PAGE and transferred into PVDF membranes, which were then incubated with primary antibodies including E-cadherin (1:1,000, ab233766, Abcam), N-cadherin (1:1,000, ab18203, Abcam), Vimentin (1:1,000, ab137321, Abcam), Hsp70 (1:1,000, ab2787, Abcam), Tsg101 (1:1,000, ab125011, Abcam), GM130 (1:1,000, ab181574, Abcam), IGF1R (1:1,000, ab182408, Abcam) and GAPDH (1:5,000, ab37168, Abcam) at 4°C overnight, followed by incubation with horseradish peroxide-conjugated secondary antibody (1:1,000) at room temperature for 2 h. Finally, the bands were observed by chemiluminescence reagent and analyzed using the ChemiDoc MP Imaging System (Bio-Rad).

### 5‐ethynyl‐2′‐deoxyuridine (EdU) staining assay

To evaluate cell proliferation, the 5‐ethynyl‐2′‐deoxyuridine (EdU) staining assay was conducted as previously described [20]. Briefly, cells were plated into 24‐well plates and cultured overnight, then 50 μmol/L EdU reagent was added into cell culture and incubated for 3 h. Cells were incubated with Hoechst 3334 at room temperature for 2 h. A fluorescence microscope was used to observe the representative images.

### Transwell assays

The invasion and migration of cancer cell lines were measured by Transwell assays using Transwell membrane coating with or without Matrigel™ (BD Biosciences) as previously described [21]. The average number of invasive and migrated cells were captured and counted under a light microscopy (Nikon, Tokyo, Japan). Magnification: × 200 in ten random fields.

### In vivo *animal model*

The animal model was constructed as previously described [22]. The posterior flank of the 12 male BALB/c nude mice (5‐week‐old) were subcutaneously inoculated with 1 × 10^7^ LNCaP cells. After the tumor grew to a volume of 100 mm^3^, the nude mice were randomly divided into 3 groups (n = 4 mice in each group): PBS group, HBMSCs-miR-NC and HBMSCs-miR-99b-5p mimics. A total of 5 × 10^5^ HBMSCs transfected with miR-99b-5p or miR-NC were injected into each nude mouse via a tail vein. The tumor volume was evaluated by L × W^2^)/2 every week for a total of 4 weeks, in which L is the length of tumor and W is the weight of tumor. Finally, mice were euthanized with CO_2_, and tumors were weighted. Meanwhile, the tumor samples were cut into 5 mm sections, fixed in 4% paraformaldehyde and embedded in paraffin, followed by immunohistochemistry assay using anti-Ki67 antibody (1:200, Abcam) as previously described [23].

### Statistical analysis

All data were presented as mean ± standard deviation (SD) using the SPSS 21.0 software. The differences were explored by Student’s t-test or one-way ANOVA between two groups or among multiple groups, respectively. *P* < 0.05 was considered as the significant threshold.

## Results

### MiR-99b-5p was downregulated in PCa tissues and upregulated in HBMSCs-derived exosomes

**Figure 1.**
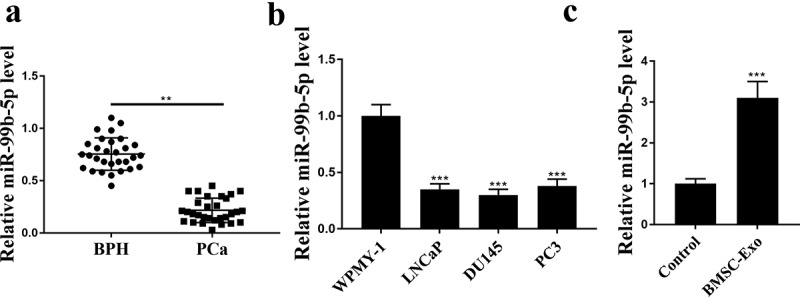
MiR-99b-5p was downregulated in PCa tissues and upregulated in HBMSCs-derived exosomes. The expression of miR-99b-5p in PCa tissues (n = 30) (a), PCa cell lines (b) and HBMSCs-derived exosomes (c) was detected by qRT-PCR. ** *p* < 0.01, *** *p* < 0.001.

To explore the role of miR-99b-5p, its expression in PCa tissues and cancer cell lines was detected by qRT-PCR. The results exhibited a significant downregulation of miR-99b-5p in PCa tissues than that in BPH tissues (*p* < 0.01, Figure 1a), as well as in PCa cell lines than that in normal WPMY-1 cells (all *p* < 0.001, Figure 1b). Interestingly, we isolated HBMSCs-derived exosomes and found that miR-99b-5p was significantly upregulated in HBMSCs-derived exosomes than that in the control group (*p* < 0.001, Figure 1c). These results suggest that miR-99b-5p may play a potential role in PCa.

### IGF1R was a target of miR-99b-5p

**Figure 2.**
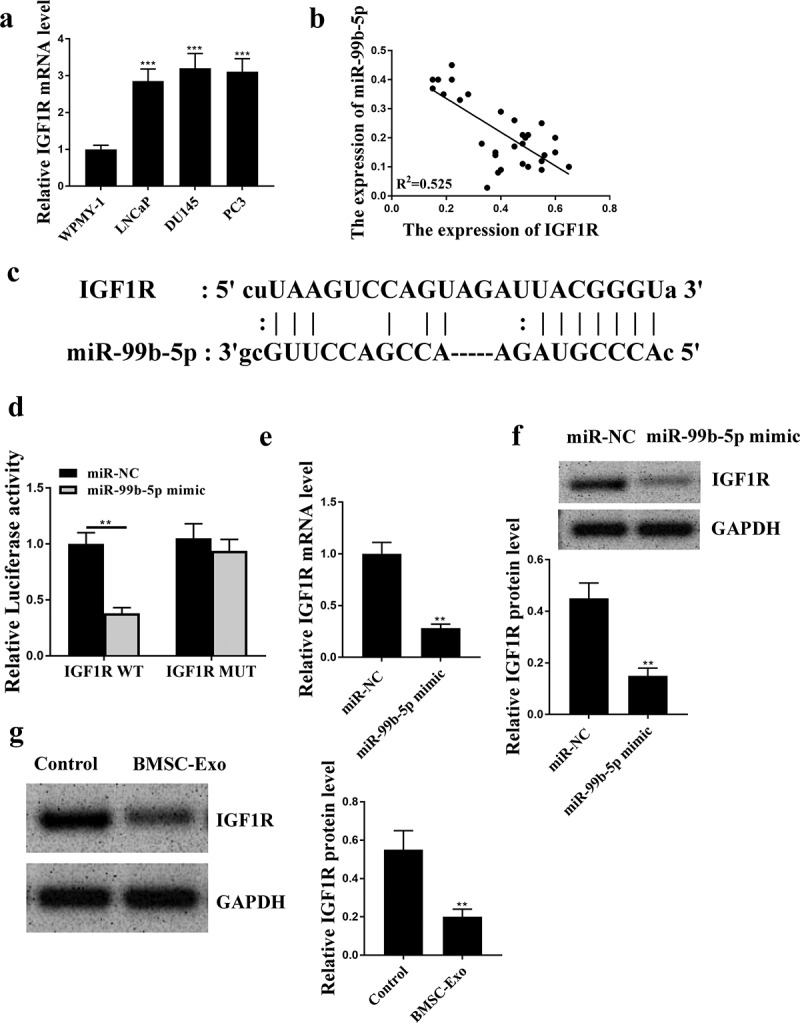
IGF1R was a target of miR-99b-5p. (a) The expression of IGF1R in PCa cell lines was detected by qRT-PCR. (b) The correlation between the expression levels of miR-99b-5p and IGF1R in PCa tissues was evaluated by correlation analysis. (c) The putative binding site between miR-99b-5p and IGF1R was predicted by Targetscan. (d) Luciferase reporter assay. (e and f) The expression of IGF1R in miR-99b-5p mimics transfected LNCaP cells was measured by qRT-PCR (e) and Western blot analysis (f). (g) The expression of IGF1R in HBMSCs-derived exosomes was detected by Western blot analysis. ** *p* < 0.01, *** *p* < 0.001.

To understand the potential regulatory axis of miR-99b-5p, the downstream target genes were investigated. qRT-PCR results showed that the expression levels of IGF1R were significantly elevated in PCa cell lines compared with that in WPMY-1 cells (all *p* < 0.001, Figure 2a). There was an obviously negative correlation between the expression levels of miR-99b-5p and IGF1R in PCa tissues (R^2^ = 0.525, Figure 2b). The putative binding site between miR-99b-5p and IGF1R was predicted by Targetscan, showing that miR-99b-5p might bind to the 3ʹ-UTR of IGF1R (Figure 2c). Luciferase reporter assay was then conducted and the results showed that miR-99b-5p mimics significantly reduced the luciferase activity of IGF1R WT in LNCaP cells compared with miR-NC group (*p* < 0.01), but had no effect on IGF1R MUT (Figure 2d). Meanwhile, miR-99b-5p mimics was transfected into LNCaP cells, and it showed that overexpression of miR-99b-5p significantly reduced the expression levels of IGF1R compared with miR-NC group (*p* < 0.01, Figure 2e and f). However, the expression levels of IGF1R in HBMSCs-derived exosomes were decreased than that in the control group (*p* < 0.01, Figure 2g). These data suggest that IGF1R is a downstream target of miR-99b-5p.

### HBMSCs-derived exosomes inhibited the progression of LNCaP cells

**Figure 3.**
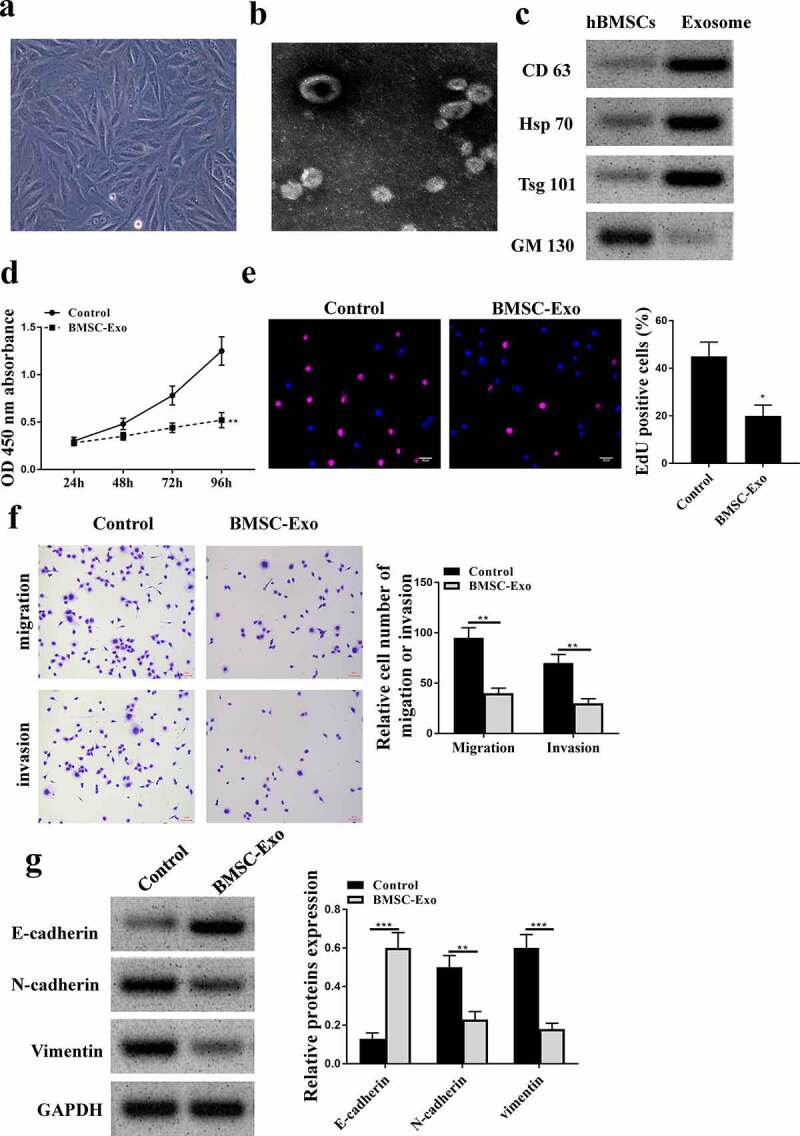
HBMSCs-derived exosomes inhibited the progression of LNCaP cells. (a) The morphological characteristics of HBMSCs by the inverted microscope. (b) The ultrastructure of exosomes by a transmission electron microscope (TEM). (c) The analysis of exosomes by Western blot to detect relative exosomes-related markers. (d-g) LNCaP cells were treated with HBMSCs-derived exosomes for different times (CCK-8 assay), EdU assay and Transwell assay (72 h). (d) The cell viability of LNCaP cells by CCK-8 assay. (e) The proliferation of LNCaP cells by EdU staining assay. Scale bar = 40 μm. (f) The migration and invasion of LNCaP cells by Transwell assay. Scale bar = 100 μm. (g) The expression of exosomes related markers was analyzed by Western blot analysis. ** *p* < 0.01, *** *p* < 0.001.

To investigate the effect of HBMSCs-derived exosomes on PCa, HBMSCs were isolated from volunteers and observed using the inverted microscope and sed to stimulate LNCaP cells. It showed that HBMSCs were large, polygonal, spindle-like, or spindle-shaped adherent cells with large nuclei in the middle of the cells (Figure 3a). Then their exosomes were extracted and the images of transmission electron microscope showed that the exosomes ranged from 40 to 100 nm in diameter, being round or dish-shaped in shape, containing low-density substances and complete lipid membrane vesicles (Figure 3b). Western blot analysis results showed that HBMSCs-derived exosomes obviously expressed exosomes-related markers (CD63, Hsp70 and Tsg101), while did not express exosome-negative marker GM130 (Figure 3c). These data suggested that HBMSCs-derived exosomes could be used for the subsequent experiments. Then a series of function experiments were conducted to investigate the role of exosomes in PCa cells, and we found that HBMSCs-derived exosomes could significantly inhibit cell proliferation (CCK-8 assay, *p* < 0.01, EdU staining assay, *p* < 0.05, Figure 3d and e), migration and invasion (both *p* < 0.01, Figure 3f) of LNCaP cells compared with that in the control group (PBS). Meanwhile, HBMSCs-derived exosomes increased the expression levels of E-cadherin (*p* < 0.001), while reduced the expression levels of N-cadherin (*p* < 0.01) and Vimentin (*p* < 0.001) in LNCaP cells compared with that in the control group (PBS) (Figure 3g). These results suggest that HBMSCs-derived exosomes inhibit the progression of LNCaP cells.

### MiR-99b-5p inhibitor transfected HBMSCs-derived exosomes promoted the progression of LNCaP cells

**Figure 4.**
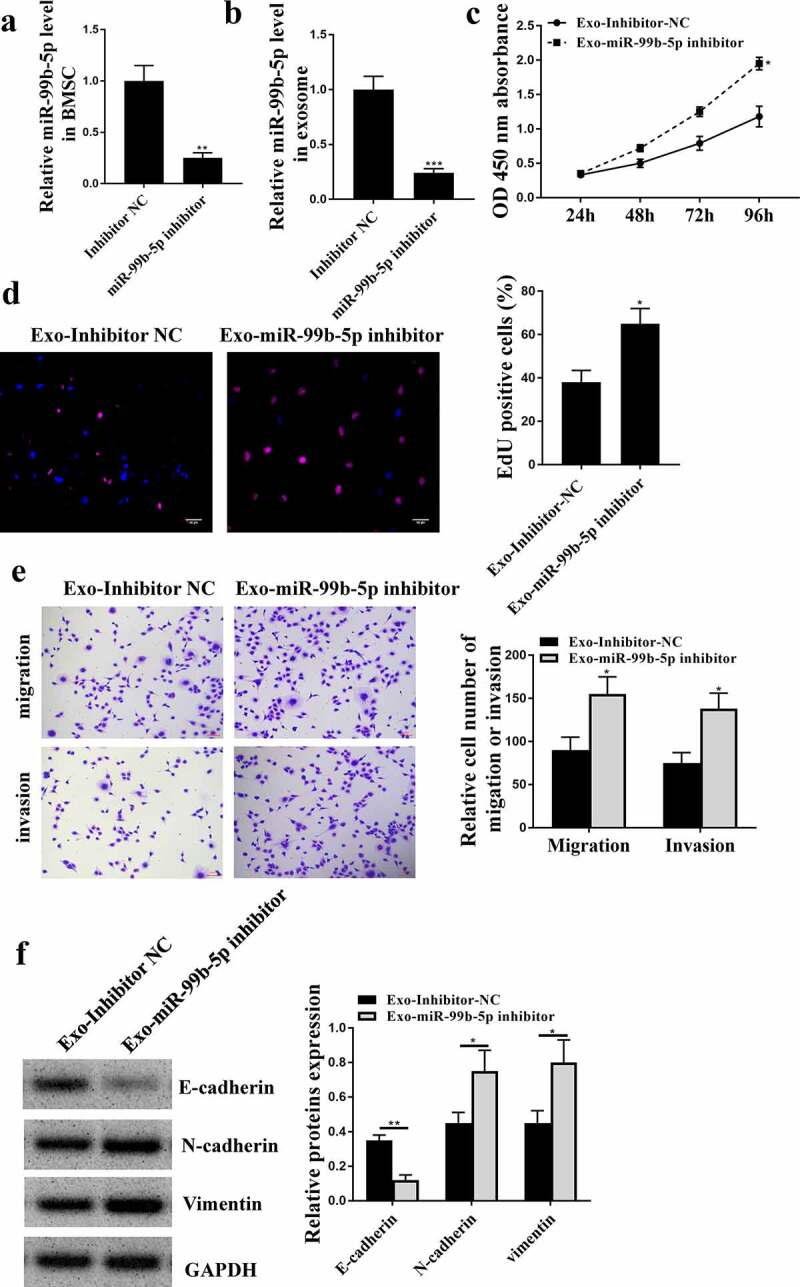
MiR-99b-5p inhibitor transfected HBMSCs-derived exosomes promoted the progression of LNCaP cells. (a and b) HBMSCs were transfected with miR-99b-5p inhibitor or inhibitor NC. The expression of miR-99b-5p in HBMSCs (a) and HBMSCs-derived exosomes (b) was detected by qRT-PCR. (c-f) 20 ug HBMSCs-derived exosomes were used to treat LNCaP cells. (c) The cell viability of LNCaP cells by CCK-8 assay. (d) The proliferation of LNCaP cells by EdU staining assay. Scale bar = 40 μm. (e) The migration and invasion of LNCaP cells by Transwell assay. Scale bar = 100 μm. (f) The expression of exosomes related markers was analyzed by Western blot analysis. * *p* < 0.05, ** *p* < 0.01.

Next, to confirm whether miR-99b-5p participated in the effects of HBMSCs-derived exosomes on the progression of LNCaP cells, miR-99b-5p inhibitor was transfected into HBMSCs and exosomes were extracted to stimulate LNCaP cells. The expression of miR-99b-5p was downregulated in transfected HBMSCs (*p* < 0.01) and HBMSCs-derived exosomes (*p* < 0.001) (Figure 4a and b). Then 20 ug HBMSCs-derived exosomes were used to stimulate LNCaP cells, and it showed that miR-99b-5p inhibitor transfected HBMSCs-derived exosomes significantly enhanced cell proliferation (*p* < 0.05, Figure 4c and d), and promoted cell migration and invasion (*p* < 0.05) of LNCaP cells compared with inhibitor NC transfected HBMSCs-derived exosomes (Figure 4e). Meanwhile, miR-99b-5p inhibitor transfected HBMSCs-derived exosomes reduced the expression levels of E-cadherin (*p* < 0.01), while increased the expression levels of N-cadherin (*p* < 0.05) and Vimentin (*p* < 0.05) in LNCaP cells (Figure 4f). These results suggest that miR-99b-5p inhibitor transfected HBMSCs-derived exosomes promote the progression of LNCaP cells.

### MiR-99b-5p mimics transfected HBMSCs inhibited the progression of LNCaP cells

**Figure 5.**
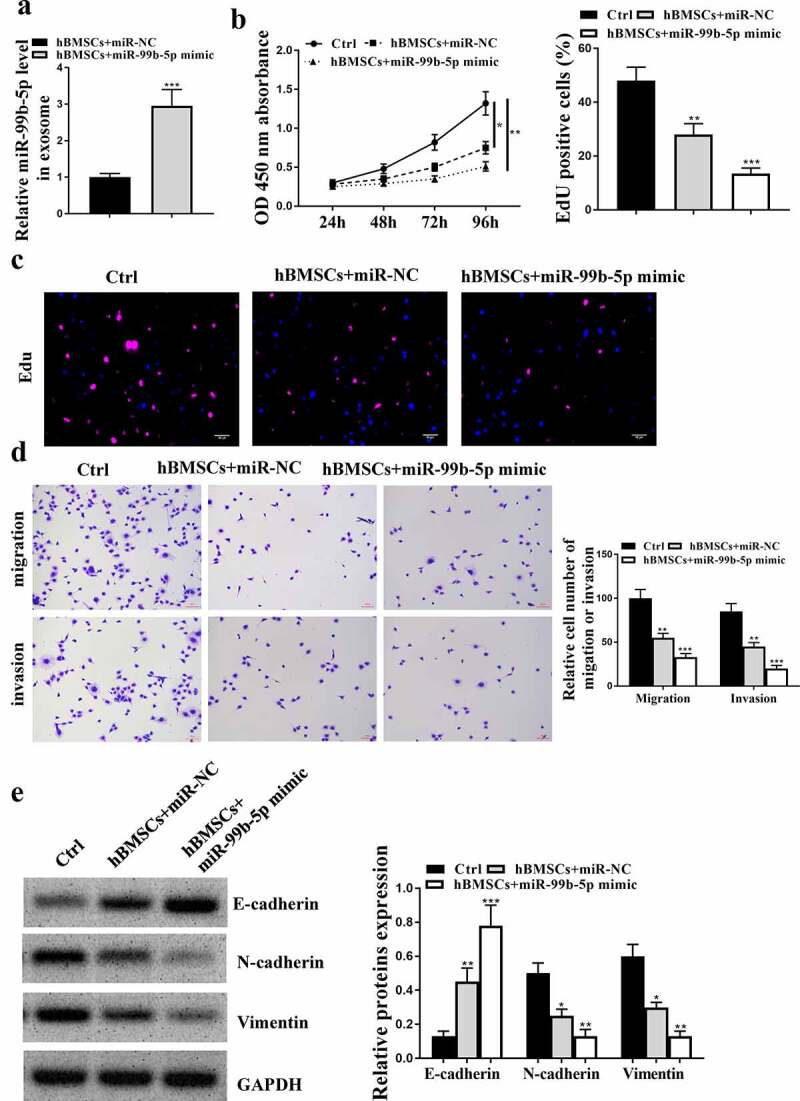
MiR-99b-5p mimics transfected HBMSCs inhibited the progression of LNCaP cells. HBMSCs were transfected with miR-99b-5p mimics or miR-NC, and transfected HBMSCs were co-cultured with LNCaP cells. (a) The expression of HBMSCs-derived exosomes was detected by qRT-PCR. (b) The cell viability of LNCaP cells by CCK-8 assay. (c) The proliferation of LNCaP cells by EdU staining assay. Scale bar = 40 μm. (d) The migration and invasion of LNCaP cells by Transwell assay. Scale bar = 100 μm. (e) The expression of exosomes related markers was analyzed by Western blot analysis. * *p* < 0.05, ** *p* < 0.01, *** *p* < 0.001.

Similarly, to confirm the role of miR-99b-5p in the effects of HBMSCs-derived exosomes against the progression of LNCaP cells, then HBMSCs were transfected with miR-99b-5p mimics, and exosomes were extracted to stimulate LNCaP cells. The expression of miR-99b-5p was significantly upregulated in miR-99b-5p mimics transfected HBMSCs-derived exosomes compared with that in miR-NC transfected HBMSCs-derived exosomes (*p* < 0.001, Figure 5a). In addition, miR-NC transfected HBMSCs significantly reduced cell proliferative rate (CCK-8 assay, *p* < 0.05, EdU assay, *p* < 0.01), and inhibited the migration and invasion (*p* < 0.01) of LNCaP cells compared with that in the control group (PBS). Moreover, miR-99b-5p mimics transfected HBMSCs further enhanced the inhibitory effects of HBMSCs on LNCaP cells (CCK-8 assay, *p* < 0.01; EdU assay, *p* < 0.001; migration and invasion, *p* < 0.001) (Figure 5b-d). Meanwhile, miR-NC transfected HBMSCs increased the expression levels of E-cadherin (*p* < 0.01), while reduced the expression levels of N-cadherin (*p* < 0.05) and Vimentin (*p* < 0.05) in LNCaP cells compared with that in the control group (PBS); while miR-99b-5p mimics transfected HBMSCs further enhanced the effects of HBMSCs on LNCaP cells (*p* < 0.001 or *p* < 0.01, Figure 5e). These results suggest that miR-99b-5p mimics transfected HBMSCs inhibit the progression of LNCaP cells.

### MiR-99b-5p mimics-transfected HBMSCs-derived exosomes inhibited tumor growth in vivo

**Figure 6.**
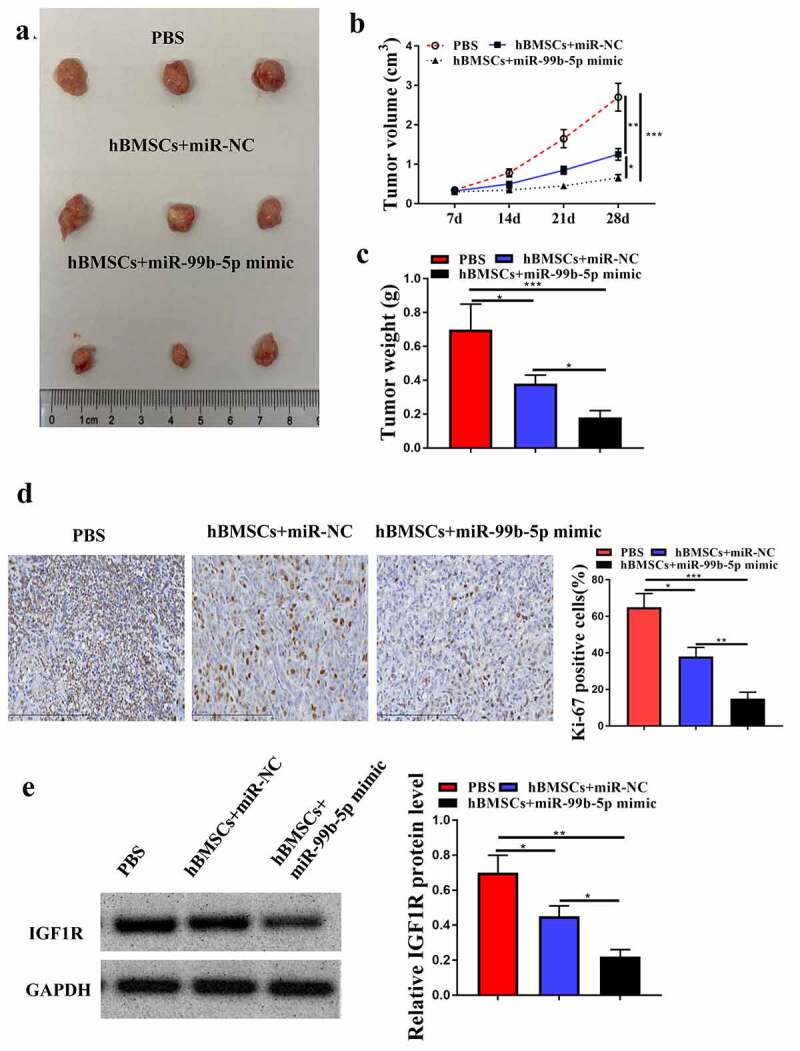
MiR-99b-5p mimics-transfected HBMSCs-derived exosomes inhibited tumor growth. (a) Tumor images. (b) Tumor volume. (c) Tumor weight. (d) Immunohistochemistry assay with anti-Ki67 antibody. (e) The expression of IGF1R in tumor tissues was detected by Western blot analysis. * *p* < 0.05, ** *p* < 0.01, *** *p* < 0.001.

Finally, to further determine the role of hBMSCs-derived exosomal miR-99b-5p in PCa progression *in vivo*, the animal model was constructed. It showed that miR-NC transfected HBMSCs obviously inhibited tumor growth, and reduced tumor volume (*p* < 0.01) and weight (*p* < 0.05) compared with PBS group; and miR-99b-5p mimics transfected HBMSCs further enhanced the inhibitory effects of HBMSCs on tumor progression (*p* < 0.05) (Figure 6a-c). Meanwhile, immunohistochemistry assay results showed that miR-NC transfected HBMSCs reduced the number of Ki-67 positive cells in tumor tissues compared with that in PBS group (*p* < 0.05), and miR-99b-5p mimics transfected HBMSCs further reduced Ki-67 positive cells compared with miR-NC transfected HBMSCs (*p* < 0.01, Figure 6d). In addition, miR-NC transfected HBMSCs decreased the expression levels of IGF1R in tumor tissues compared with that in PBS group (*p* < 0.05), and miR-99b-5p mimics transfected HBMSCs further reduced the expression levels of IGF1R compared with miR-NC transfected HBMSCs (*p* < 0.05, Figure 6e). These results suggest that miR-99b-5p mimics-transfected HBMSCs-derived exosomes inhibit tumor growth by downregulating IGF1R.

## Discussion

In the past decades, increasing attentions have been focused on PCa due to its serious health burden in men [24]. In recent years, exosomes mediated therapies have been considered as a promising therapeutic approach because of their abilities of being naturally taken up by cells, and capable of the stable transfer of drugs, therapeutic miRNAs and proteins [25]. In the present study, we found that miR-99b-5p was downregulated in PCa and upregulated in HBMSCs-derived exosomes. Moreover, HBMSCs-derived exosomes with overexpression of miR-99b-5p could obviously attenuate the development of PCa, indicating that HBMSCs-derived exosomes might be a promising therapeutic strategy for PCa.

The dysregulation of miRNAs in PCa was always observed, and more of them have been well studied, contributing to our knowledge of PCa development [26]. For example, miR-215-5p was reported to suppress the metastasis of prostate cancer, and also closely associated with the prognosis of PCa patients [27]. Recently, the potential functions of exosomes and exosomal miRNAs in biologic processes of PCa progression and radiation therapy response have been studied, revealing that exosomes might contribute to cancer therapy by regulating miRNAs [28]. In this study, our data demonstrated that miR-99b-5p was significantly upregulated in HBMSCs-derived exosomes, which confirmed the findings that miR-99b-5p was enriched in serum exosomes of PCa patients treated with radiotherapy [11]. Meanwhile, HBMSCs-derived exosomes could attenuate the progression of PCa, and HBMSCs-derived exosomes with the overexpression of miR-99b-5p further enhanced the protective effects of HBMSCs-derived exosomes, suggesting that HBMSCs-derived exosomes carrying drugs that increased the expression levels of miR-99b-5p and even only HBMSCs-derived exosomes might be used for treat PCa. However, how to delivery HBMSCs-derived exosomes to human with no harm is still a challenge and needs to be explored well.

Based on the important roles of exosomal miR-99b-5p in PCa, we explored its potential underlying mechanisms. Through bioinformatics analysis and luciferase reporter assay, we identified that IGF1R was a target of miR-99b-5p, and there was a negative correlation between the expression levels of miR-99b-5p and IGF1R in PCa tissues. Previous studies demonstrated that IGF1R was upregulated in PCa tissues and high expression levels of IGF1R was positively correlated to the aggressive phenotypes of PCa cells [29–31]. Interestingly, Wang *et al*. revealed that miR-99b-5p could regulate the development of gastric cancer by targeting IGF1R [13], suggesting that IGF1R was a target of miR-99b-5p. As expected, we demonstrated that miR-99b-5p could bind to the 3ʹ-UTR of IGF1R, and overexpression of miR-99b-5p reduced the expression levels of IGF1R in LNCaP cells. Meanwhile, the downregulation of IGF1R in HBMSCs-derived exosomes also verified the inhibitory impacts of exosomal miR-99b-5p on PCa progression. Except for IGF1R, several other downstream genes of miR-99b-5p have been reported in previous studies, such as FGFR3 [32] and MFG-E8 [33]. Whether these downstream genes mediated the function of exosomal miR-99b-5p in PCa needs to be studied in the future.

Although our *in vivo* results revealed that HBMSCs with overexpression of miR-99b-5p obviously inhibited tumor growth and downregulating the expression of IGF1R, the manipulation of IGF1R *in vivo* should also be explored to confirm the conclusion.

## Conclusion

In summary, our results revealed that HBMSCs-derived exosomes might attenuate the progression of PCa through exosomal miR-99b-5p mediated suppression of IGF1R, suggesting that HBMSCs-derived exosomes might be a potential therapeutic strategy for PCa.

## Data Availability

The data was available from the corresponding author upon reasonable request.

## Approval of the research protocol by an Institutional Reviewer Board

This study was approved by the Haikou Hospital of Traditional Chinese Medicine.

## List of abbreviations

PCa: Prostate cancer; HBMSCs: Human bone marrow mesenchymal stem cells; EMT: Epithelial-mesenchymal transition
